# Nanoparticle Targeting with Antibodies in the Central Nervous System

**DOI:** 10.34133/bmef.0012

**Published:** 2023-03-31

**Authors:** Ju Hyun Lee, Dana V. Chapman, W. Mark Saltzman

**Affiliations:** Department of Biomedical Engineering, Yale University, New Haven, CT, USA.

## Abstract

Treatments for disease in the central nervous system (CNS) are limited because of difficulties in agent penetration through the blood-brain barrier, achieving optimal dosing, and mitigating off-target effects. The prospect of precision medicine in CNS treatment suggests an opportunity for therapeutic nanotechnology, which offers tunability and adaptability to address specific diseases as well as targetability when combined with antibodies (Abs). Here, we review the strategies to attach Abs to nanoparticles (NPs), including conventional approaches of chemisorption and physisorption as well as attempts to combine irreversible Ab immobilization with controlled orientation. We also summarize trends that have been observed through studies of systemically delivered Ab–NP conjugates in animals. Finally, we discuss the future outlook for Ab–NPs to deliver therapeutics into the CNS.

## Introduction

Pathologies in the central nervous system (CNS)—ranging from vascular diseases to neurodegenerative diseases to tumors—are a leading cause of disability in the world, and their prevalence has increased in the 21st century [1]. Developing effective treatments for CNS disorders involves three interconnected considerations: moving agents across the blood-brain barrier (BBB), achieving an effective dose at the cellular target, and limiting accumulation in off-target cellular populations. The BBB is the main structural obstacle in developing CNS therapies; nearly 98% of systemically delivered, small-molecule drugs are unable to enter the brain [2]. For those that do penetrate the BBB, the realized concentration of drug in the CNS is much less than the systemic concentration [3]. For this reason, effective dosing in the CNS often requires high systemic concentrations that produce toxicity in other tissues. Finally, there must be a way to mitigate off-target delivery of therapeutics in the CNS, as neurological complications can be detrimental [4].

Nanotechnology is a promising approach to solving these three challenges. Garnering interest and activity since 1959, the year of Richard Feynman’s notable lecture [5], the idea of small, tunable nanomaterials has been realized for applications in a wide range of fields, from electronics to medicine [6]. Early evidence suggested that intrinsic properties of certain materials—such as size and surface charge—can be optimized for preferential accumulation in the CNS. For example, nanoparticles (NPs) between 10 and 100 nm in diameter, which were theoretically small enough to permeate the BBB (<200 nm) [7–9] yet large enough to avoid rapid clearance from the circulation by renal filtration (>5 nm), were discovered to be optimal [10]. Slightly cationic NPs have also been used to avoid toxic effects from strongly cationic NPs [11,12], while still leveraging electrostatic interactions with the BBB’s anionic surface to facilitate their crossing. For example, polyamidoamine dendrimers that are hydroxylated—such that the initial cationic charge of the dendrimers shifts to near-neutrality—can cross the BBB, with an improvement in circulation half-life and reduction of toxicity [13,14].

Outside of the CNS, NPs have an inherent, passive targeting capacity for solid tumors, due to the enhanced permeability and retention (EPR) effect [15]. Modifying NP size or surface moieties [e.g., polyethylene glycol (PEG)] can reduce premature clearance or off-target uptake, allowing them to achieve sufficiently high accumulation at tumor sites to induce therapeutic effects [16]. However, this passive targeting faces clear limitations when the pathophysiology of a disease does not provide the leaky vasculature and impaired lymphatic drainage that facilitates the passive accumulation of NPs in tumors [15]. In general, systemically delivered NPs do not accumulate in brain tumors. Despite hopes that a defective BBB might facilitate the EPR effect, most studies in animals with intracranial tumors reveal that less than 1% of systemically delivered NPs reach the brain or tumor [17–20]. Enhancing BBB permeability using focused ultrasound (FUS) [21] can enhance penetration after systemic administration. Another way to overcome this obstacle is to change administrative routes: Rather than systemic administration, convection-enhanced delivery (CED) [22], intrathecal delivery [23], and nasal delivery approaches [24] have demonstrated improved NP uptake and reduced systemic toxicity.

**Fig. 1.**
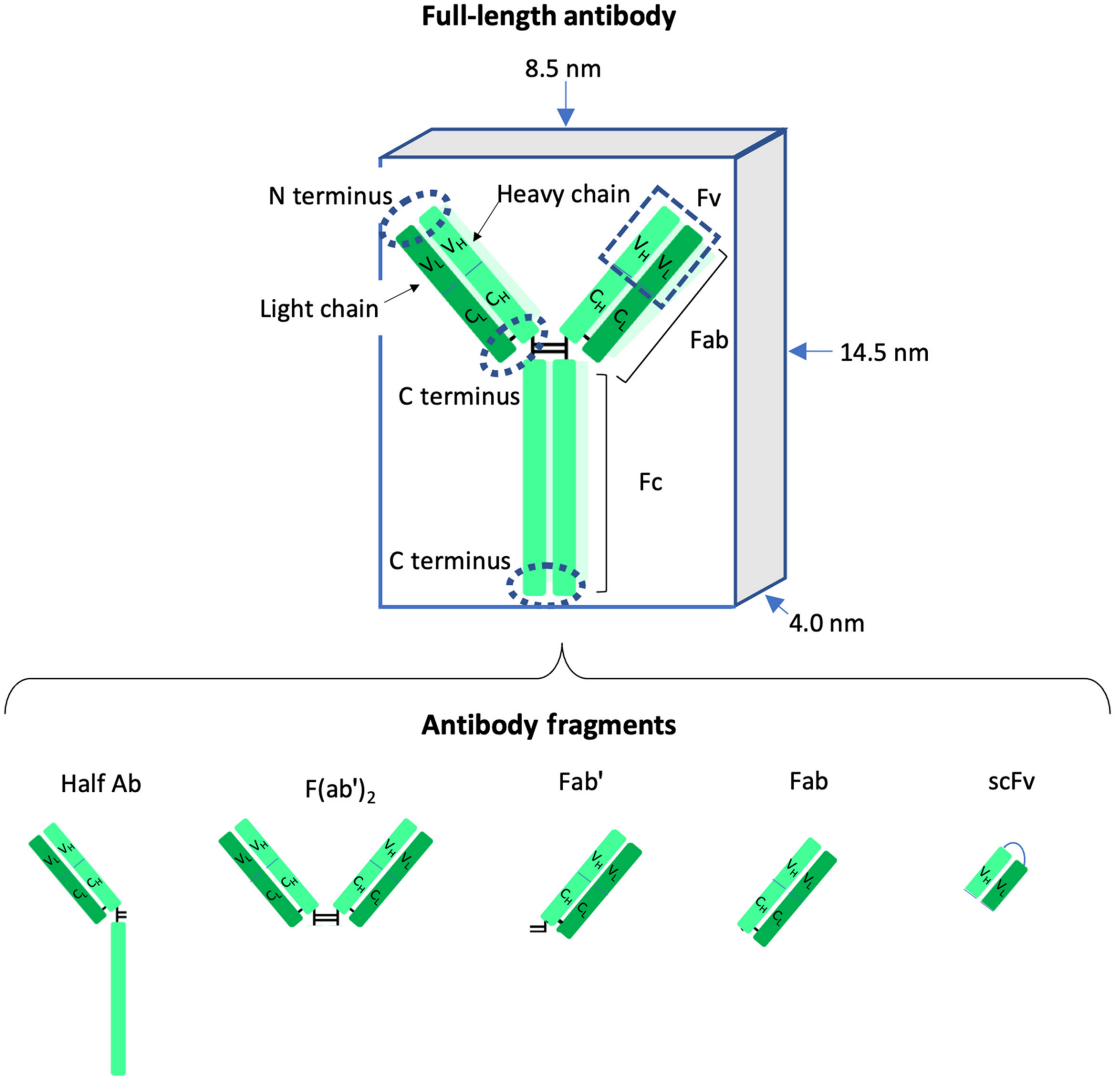
Simplified representation of the IgG1 Ab, both full-length (top) and fragment (bottom). Heavy chain is represented with light green, and light chain is represented with dark green; other aspects of the Ab, as discussed in the main text, are denoted. Locations of N and C termini are designated by dotted ovals and labeled. Typical dimensions of the Ab are labeled. Key disulfide bonds in the Ab structure are represented by the black lines, which when broken (such as in Ab fragments) lead to further available thiols for conjugation to NPs.

Alternatively, incorporating a targeting moiety, such as an antibody (Ab), onto the surface of NPs can enhance penetration by taking advantage of specific transport pathways through the BBB. Further, Ab-conjugated NPs (Ab–NPs) can result in increased retention by targeted cell populations, and they can also enhance uptake into specific cells through receptor-mediated endocytosis [10,25]. Often misleadingly characterized as “active” targeting—to differentiate it from “passive” targeting due to biological features of the tissue, such as the EPR effect—Ab targeting does not enhance homing to cell populations but rather increases NP retention at targeted sites. Many Abs can be attached to an NP surface: For example, immunoglobulin G (IgG) is about 10 nm in diameter (Fig. 1), and NPs are typically 100 to 200 nm in diameter. Therefore, an Ab–NP can take advantage of multivalency, which enhances the likelihood of desirable interactions between Ab–NPs and a specific cellular population [26].

Abs are glycoproteins that demonstrate high binding specificity to antigens. In addition to their natural role in facilitating humoral immune responses against pathogens or toxins [15], Abs have also been an important early example of precision medicine, as genetic or molecular profiling informed their use [27]. Over the years, a number of Abs have been approved as treatments for human use, such as cetuximab, trastuzumab, and others that have been listed comprehensively elsewhere [28,29]. Through modern methods of protein engineering, Abs can be modified to elicit minimal immune responses while maintaining high antigen-binding specificity [30]. Among the many classes of Abs, IgGs have been most extensively explored for therapeutic applications. Composed of two light chains and two heavy chains stabilized by several disulfide bonds, IgGs are about 10 nm in a typical dimension (14.5 nm × 8.5 nm × 4 nm, to be more specific [31]). The structure of IgGs can be functionally divided into two components: the fragment antigen-binding portion (Fab portion) and the fragment crystallizable portion (Fc portion) (Fig. 1). The Fab can be further broken down into variable (Fv, V_H/L_) and constant (C_H/L_) regions, the former being responsible for antigen recognition. Each IgG displays two Fab regions, so they are bivalent; two antigen-binding sites are available for binding per Ab. Specific sections of Abs can furthermore be isolated or engineered, to create smaller Ab fragments that retain antigen recognition [32].

**Fig. 2.**
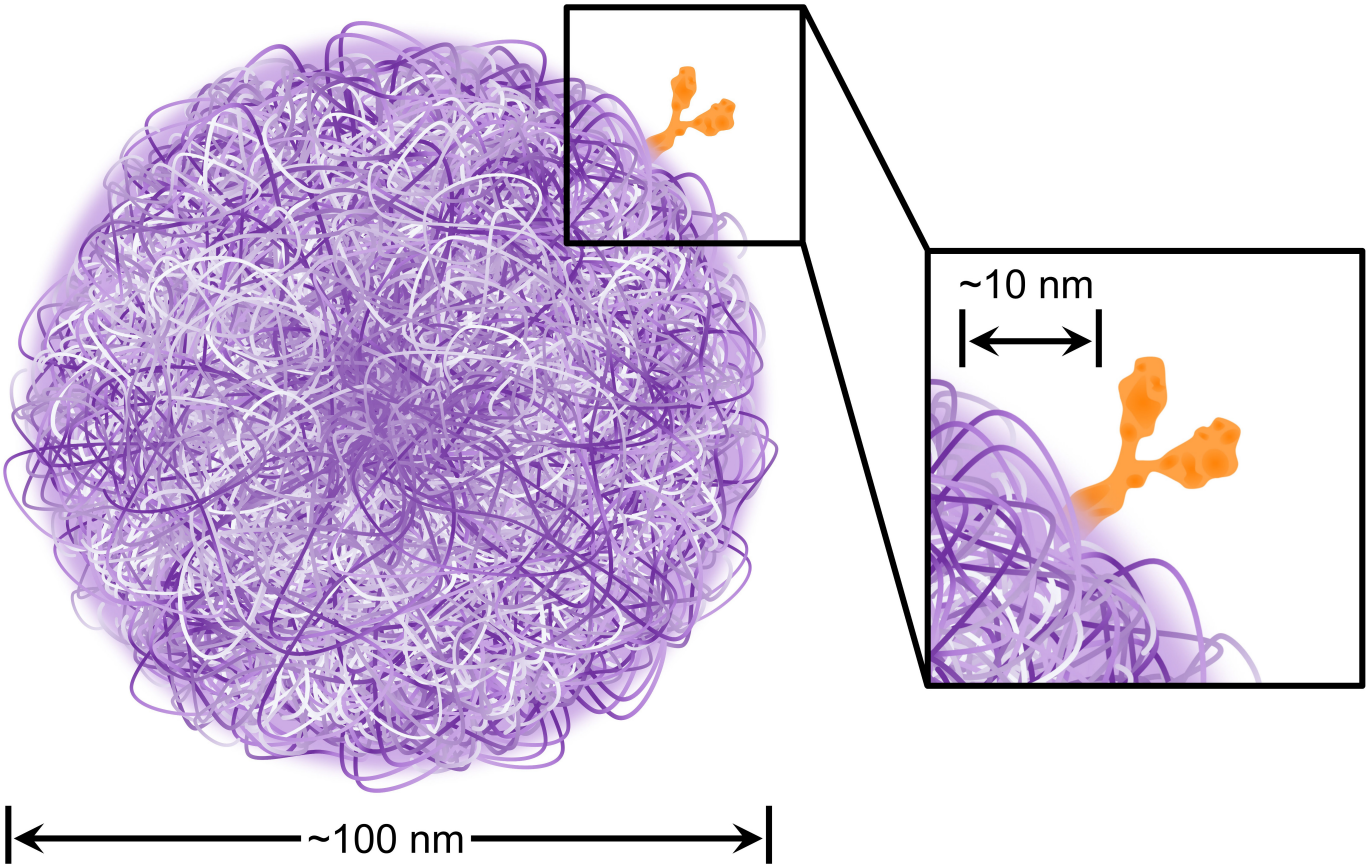
Scaled representation of a single Ab conjugated to an NP composed of polymer chains. In general, many Abs are coupled to the surface of a 100-nm NP. Notice that the surface of the polymer NP is not smooth but can sometimes be controlled through polymer synthesis and/or NP assembly (see Grundler et al. [93] for example).

While many of the currently approved Abs act by preventing a biological interaction—such as Abs that act as checkpoint inhibitors by blocking the interaction of programmed cell death protein 1 and programmed death-ligand 1 [33]—some Abs are engineered to deliver a drug payload. The idea of combining Abs with a therapeutic agent—in the form of antibody-drug conjugates (ADCs) [34]—has opened a new door for the development of targeted agents. Ab–NPs have several potential advantages over small-molecule ADCs: increased drug loading, multivalency, and versatility through Ab and NP selection and conjugation strategy. Despite high localization to targeted cellular populations, ADCs suffer from a low drug-to-Ab ratio (generally two to four). Ab–NPs can address this shortcoming, as they can carry up to 10 times this amount without increasing risk of toxicity [35]; Figure 2 shows a to-scale representation of a polymer-based Ab–NP. Furthermore, Ab–NPs are multivalent in several respects. Like ADCs, Ab binding can prolong contact with cell surfaces and thus promote internalization of the Ab–NP and its payload. However, even Ab–NP retention to the cell through Ab binding leads to localized release of therapeutic payload. In addition, one Ab–NP can display multiple Abs, which further increases the possibility of adhesion to the surfaces of targeted cellular populations as compared to ADCs. Lastly, while ADCs tend to incorporate disease-specific Abs for localization and safety, Abs for NP conjugation can be selected to leverage existing mechanisms to overcome physiological obstacles, such as crossing the BBB through receptor-mediated transcytosis [10]. These multiple benefits of Ab–NPs showcase their potential as candidates for treating disease in the CNS. In this review, we present an overview of Ab conjugation techniques for NPs. We also discuss important design concepts and strategies for developing Ab–NPs that target the CNS.

## Methods for Ab–NP Conjugation

**Fig. 3.**
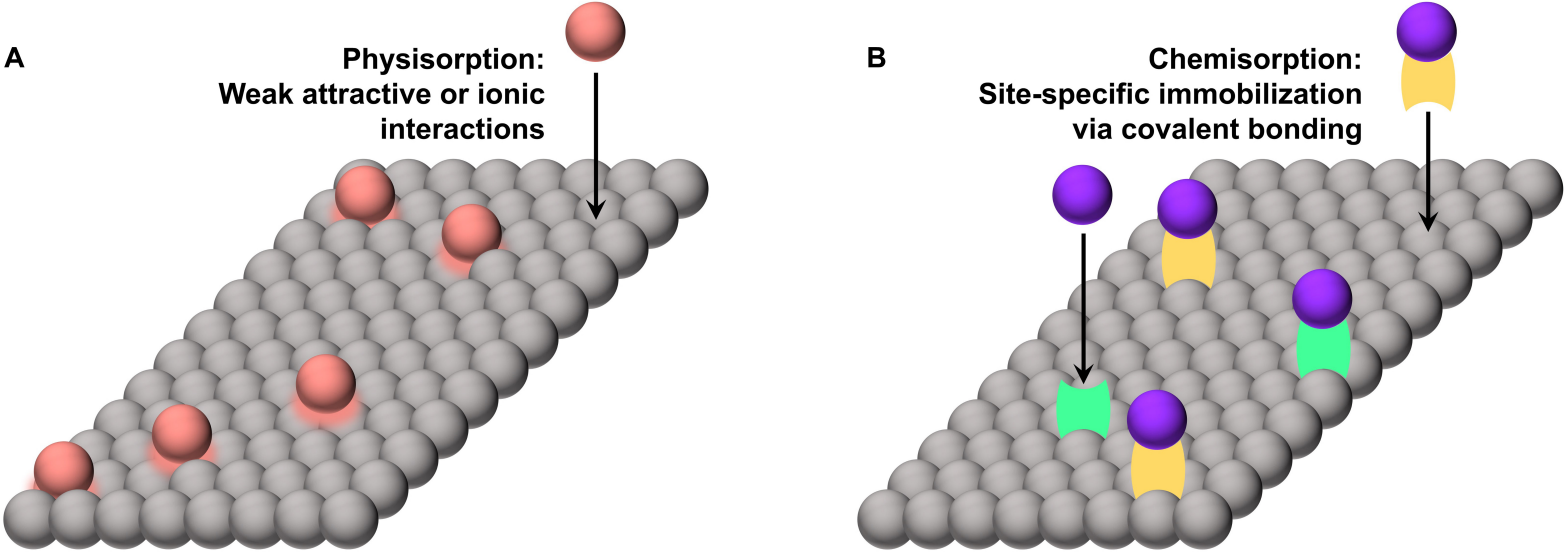
Simplified representation of the difference between physisorption (A) and chemisorption (B) of objects (red and purple spheres, respectively) to a substrate surface (layer of gray spheres). Chemisorption often uses chemical modification of an object (yellow linker) or functionalization of the surface (green linkers) to achieve covalent attachment.

**Fig. 4.**
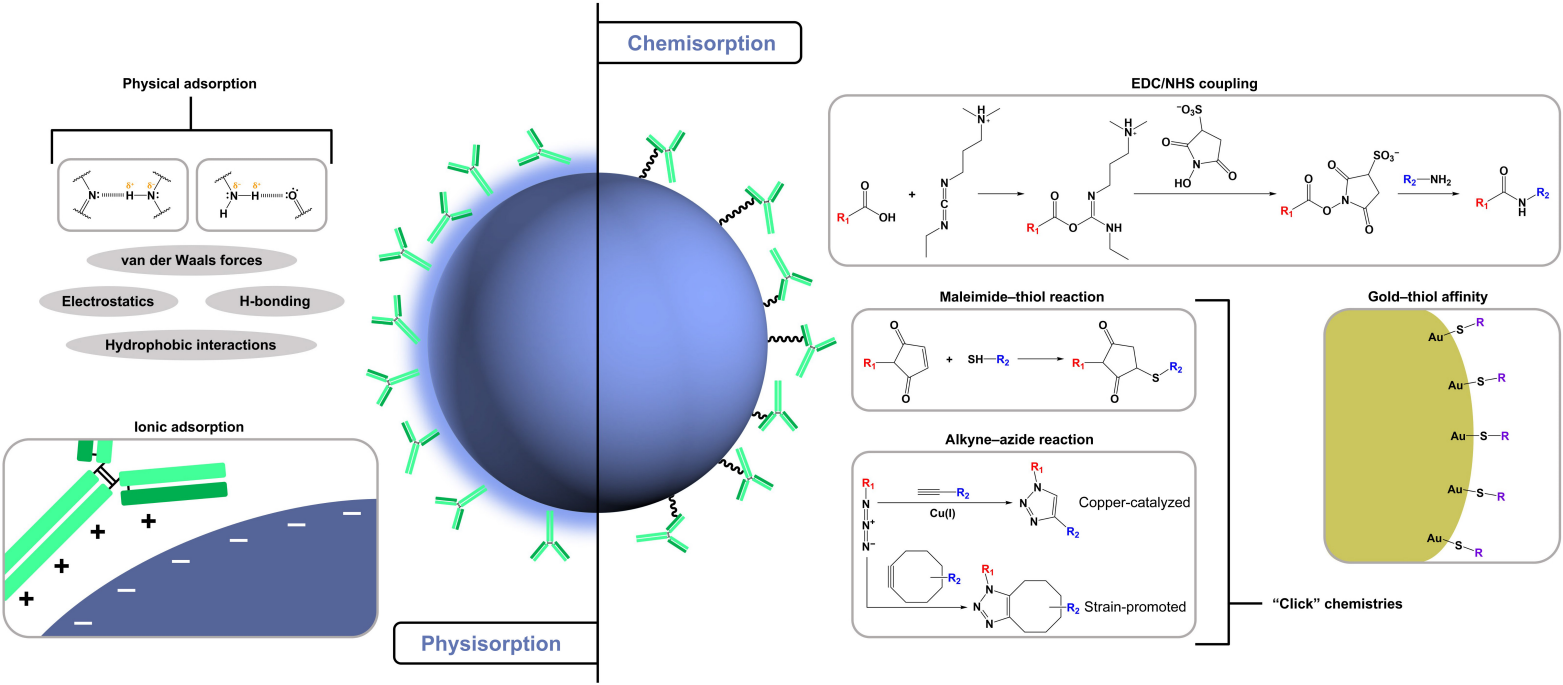
Representative examples of physisorption- and chemisorption-based conjugation methods. Physisorption (left) takes advantage of nonspecific Ab–NP interactions and encompasses physical adsorption, mediated by weak attractive forces, and ionic adsorption, mediated by charge differences. Chemisorption (right) uses chemical reactions to actively attach Ab moieties to functionalized NP surfaces, commonly via EDC/NHS coupling, maleimide–thiol or alkyne–azide “click” chemistries, or gold–thiol affinity.

Strategies for attachment of Abs to NPs can be broadly divided into two classes: physisorption and chemisorption (Fig. 3) [36]. Physisorption, or physical adsorption, facilitates noncovalent attachment of Abs to NP surfaces based on weak attractive forces—electrostatic, hydrophobic, hydrogen bonding, and van der Waals interactions [37]. Physisorption also encompasses ionic binding, whereby opposite charges on Ab moieties and NP surfaces mediate their association [15]. While this adsorption strategy is convenient and often displays orientational control over surface-conjugated Abs [38], it is also reversible, which renders it sensitive to environmental conditions and susceptible to Ab displacement by other ligands [39]. Chemisorption, on the other hand, uses reactive chemical conjugation between Ab and NP moieties to produce stable covalent bonds [36]. While chemisorption provides robust methods of irreversible attachment, it frequently suffers from tedious preparation and uncontrolled Ab orientation [37]. Figure 4 illustrates common examples of each conjugation strategy, and Table summarizes the general advantages and disadvantages of different methods. Recent efforts have sought to combine physisorption and chemisorption to take advantage of their Ab orientational control and coronal stability, respectively [38]. The mode of conjugation frequently determines ligand density and orientation [40], which, in addition to other factors such as NP shape, size, and material [41], have direct influences over Ab–NP performance [40,42,43].

**Table.** Summary of general characteristics of physisorption- and chemisorption-based approaches for Ab–NP conjugation.

**Mode of conjugation**	**Specific method**	**Advantages**	**Disadvantages**
Physisorption	Physical adsorption (weak attractive forces)	Orientational control (association via Fc portion)	Chance of displacement by serum proteins, reduced reproducibility
	Ionic adsorption	Rapid association of Ab with NP surfaces	Reversibility, vulnerability to charge environment
Chemisorption	EDC/NHS coupling	Direct covalent coupling, widely used in bioconjugation	Heterogeneous surface modification, random orientation
	“Click” chemistry	Superior covalent binding strength (compared to EDC/NHS coupling), increased reaction specificity, high yields	Chemical modification of Ab or NP surface often required
	Gold–thiol affinity	Resistance to displacement, better orientational control than other covalent bioconjugation techniques	Limitation to gold-based NPs

### Physisorption-based approaches

Noncovalent Ab–NP physisorption is easy to implement and preserves antigen-binding efficacy, although exhibiting lower bond strength and reproducibility than covalent anchoring [30,44]. Physical adsorption is widely used to couple Abs to NP surfaces [45–47] because it avoids multistep preparations, chemical modifications, and reliance on reactive linkers [43,48,49]. One factor that complicates noncovalent bioconjugation of Abs to NPs is the protein corona, a layer of proteins that associate with nanocarrier surfaces within seconds upon NP introduction to protein-rich environments [50–52]. Because of its dynamic nature, the protein corona begins with loose attachment of abundant local proteins that gradually undergo displacement by proteins with higher affinities [53], further evolving in response to changes in the surroundings [54]. As a result, proteins in biological media may supplant physically adsorbed Abs more readily than Abs covalently anchored onto the surfaces of NPs [52]. Abs that are physically adsorbed onto NP surfaces thus face considerable challenges in practice from competing interactions and sensitivity to pH, temperature, and ionic strength [15,30,38,52,55]. Dynamic changes, e.g., in the tumor microenvironment [56], therefore represent a major obstacle in adapting physisorbed Ab–NPs for real-world scenarios.

Adapters on the NP surface can facilitate the physisorption of Abs. As the strongest noncovalent interaction in biology, with stability and specificity several orders of magnitude greater than Ab–antigen affinities, biotin–(stept)avidin is frequently exploited in noncovalent Ab conjugation [57]. For Ab–NPs, researchers often use biotin-modified or “biotinylated” Abs conjugated to avidin-bearing NP surfaces [37]. For example, avidin–lipid conjugates adsorbed onto poly(lactic-*co*-glycolic acid) (PLGA) NPs were used to demonstrate a positive correlation between lipid hydrophobicity and incorporation of avidin on the NP surface [58]. Functionally, PLGA NPs bearing optimized avidin–linoleic acid conjugates showed a 13× increase in biotin-binding ability, as compared to NPs prepared with unconjugated avidin (i.e., sans fatty acid). The increase in avidin ligand density improved conjugation to biotinylated Abs and, by extension, NP targeting of CD4^+^ T cells. To evaluate relative strength of biotin–streptavidin (i.e., bond stability) based on positional interaction, unbinding forces were calculated on the basis of different geometric conformations [59]. The increased complexity of a nanoscale regime, where surfaces are no longer locally planar, produced a range in the magnitude of unbinding forces of over four-fold, depending on attachment geometry. Regardless of this variability, use of biotin–(strept)avidin affinity consistently produces accessible ligands with high bioactivity for cellular targeting [42,58,60–62], including delivery across the BBB [57].

### Chemisorption-based approaches

Covalent immobilization of Abs onto NP surfaces is characterized by stability, environmental independence, reproducibility, and adaptability [15,30]. To accomplish chemical attachment, cysteine and lysine residues are often exploited for their functional side chains, which bear reactive thiol and primary amine moieties, respectively [38]. Lysine residues are present on the N-termini of Fab portions, rendering the antigen-binding sites on Abs frequent locations for many covalent conjugations and preventing external presentation of these regions [46]. As a result, this set of methods often results in inefficient surface ligand packing (i.e., random Ab orientation) and a decrease in antigen-binding activity [36]. Alternatively, polysaccharides in the Fc region may undergo periodate-based oxidation to produce amine-reactive aldehyde moieties [30,38]. Covalent immobilization typically requires chemical modification of the biological and synthetic agents involved, which may take numerous preparation steps and can influence NP quality, particularly if an active agent is encapsulated in the NP before Ab conjugation. Still, a major advantage of chemisorption-based Ab–NP conjugation over physical adsorption is resistance to displacement of the desired ligands by local serum components [30].

A prevailing chemisorption tool is the coupling reaction of 1-ethyl-3-(3-dimethylaminopropyl)carbodiimide (EDC) and *N*-hydroxysuccinimide (NHS), which facilitates the formation of a stable amide bond between carboxylic acids and primary amines (Fig. 4). EDC serves as a zero-length cross-linker, facilitating direct covalent coupling without incorporating into the final product structure, and NHS helps to stabilize the intermediate, thus resulting in improved reaction efficiency [15]. Without NHS, EDC chemistry is prone to side reactions and Ab self-polymerization that hinders targeting ability [44]. EDC/NHS chemistry has been used in both organic [63–66] and inorganic [67–69] nanocarriers for the conjugation of Abs. However, EDC/NHS coupling also has its disadvantages: In particular, the relative nonspecificity of carbodiimide chemistry leads to heterogeneous surface modification with random orientation of Abs [36,70].

EDC/NHS coupling via stable amides is well studied and widely used in bioconjugation; however, there are other reliable approaches for covalent attachment of Abs to NP surfaces. “Click” chemistries (Fig. 4) represent a class of robust reactions characterized by stereospecificity and high yields with low or negligible by-products, which proceed under mild conditions [71]. Several groups have explored maleimide–thiol [46,65,68,70,72], Diels Alder [48,73], or alkyne–azide [43,49,74] click reactions for conjugation of Abs to NPs; one study demonstrated an eight-fold enhancement in Ab–NP binding capability in breast cancer cells compared to those prepared via EDC/NHS coupling [43]. This enhancement was attributed to functionalization of the Fc region and NP surfaces via click chemistries, which resulted in oriented immobilization. In particular, click-type alkyne–azide reactions are highly site-specific [49]—a major advantage in stable Ab immobilization. Another interaction that falls into the category of chemisorption is that between gold and sulfur, which has been used regularly to modify gold NPs with Abs [55,75–77]. These conjugates are associated with resistance to displacement [52] and controlled Ab orientation upon immobilization [78]. The packing that typifies gold–thiol interactions in two dimensions can further translate to orientational and/or positional ordering of chains on curved NP surfaces [79].

### Combined approaches

In general, physical adsorption of Abs onto nanocarrier surfaces is associated with reversibility and poor reproducibility, whereas random orientation and reduced Ab bioactivity plague covalent immobilization [30]. Several efforts have directly compared physisorption- and chemisorption-mediated Ab–NP conjugation for the same NP system. Tonigold et al. [80] reported that physical adsorption of Abs onto magnetic polystyrene NPs before plasma exposure preserved some cellular targeting and uptake, while covalently immobilized Abs were virtually completely masked by the protein corona. A theoretical treatment based on minimizing free energy indicated that covalent conjugation is necessary for antigen capture at low antigen concentrations [40]. Oliveira et al. [47] observed that on gold NPs, while physisorption corresponded to higher rates of Ab binding, chemisorption exhibited a higher relative degree of Fc-based attachment, with ~85% of conjugated Abs exhibiting oriented immobilization versus ~38% for physically adsorbed Abs. There is also evidence that differences in Ab immobilization based on conjugation protocol may be less stark than previously believed. Di Nardo et al. [39] found that immunoassay sensitivity depended more on the amount of bound Ab than the actual method of conjugation to gold NPs. Zhang et al. [76] showed that indirect assays can overestimate Ab coverage on gold NP surfaces by three- to four-fold: Enzyme-linked immunosorbent assays falsely report physisorption-based binding as substantially higher than that achieved via chemisorption-based binding, despite giving comparable values by direct fluorescence detection. Detection reliability could therefore account for some discrepancies comparing surface coverages of NPs with Abs by different conjugation methods.

Given such uncertainty, it may be best to combine the advantages associated with both approaches: preservation of targeting ability, reduction in nonspecific binding, oriented Ab immobilization, and resistance to environmental changes [38,56,81]. Gao et al. [38] have described a “bait-and-hook” approach, where Abs first physically adsorb onto NP surfaces with optimal orientation, followed by covalent reactions to affix the ligands. The “bait” encompasses Ab attachment based on preadsorption via metal affinity or ionic interactions with the Fc region, while photocrosslinking or reactive covalent coupling mechanisms anchor the adsorbed moieties. Such methods take advantage of discrepancies in relative binding rates, e.g., how much more rapid reversible ionic adsorption is than covalent reactions [36]. In one example, Parolo et al. [75] took advantage of interactions between negatively charged gold NP surfaces and an abundance of positive charges along the Fc region before fixing the desired Ab surface orientation via EDC/NHS coupling. While some studies have focused on these combined approaches in NPs [82,83], most have used two-dimensional substrates as model systems [38].

Linker or spacer systems for oriented immobilization of Abs also make use of combinatorial conjugation approaches [42,58,59,84]. The presence of such connecting chains generates interesting effects, revealing insights into the relationship between conjugation and targeting ability. In polystyrene NPs, the use of a PEG spacer likely reduced nonspecific binding of cells: Enhancing steric access of maleimido groups on NP surfaces to thiol-bearing Fc fragments facilitated the chemisorption of half Abs. In turn, these half Ab–NPs demonstrated a 1.65× improvement in targeting of CD44v6-expressing gastric cancer cells over the unconjugated PEGylated NPs [72]. In quantum dots (QDs) covalently conjugated to Abs via direct EDC/NHS coupling or to half Abs using heterobifunctional PEG spacers, the latter considerably reduced nonspecific binding despite lower Ab–QD labeling efficiency [68]. Yong et al. [42] optimized PEG lengths between single-domain Abs and QD surfaces to show how flexibility influences targeting efficiency; a longer spacer, for instance, allowed otherwise randomly oriented Abs to adopt more favorable binding conformations. These observations are consistent with theoretical models incorporating spacers based on PEG units. Regardless of affinity, maximum antigen capture increased markedly when a spacer separated the Ab and NP surface, although the effect was more pronounced at higher antigen concentrations. This effect was attributed to greater binding volume (i.e., available spaces for opportunistic antigen–Ab interactions) due to increased conformational freedom. Interestingly, the incorporation of a 50-monomer spacer appeared more beneficial for chemisorption-based conjugation than for physisorption modeled after biotin–streptavidin interactions, likely due to different mechanisms of chain reorganization on crowded NP surfaces [40]. Use of spacers may even help to preserve bioactivity of Abs [47] or Ab fragments [68], a reported drawback of covalent conjugation to NPs [36,44,51]. Taken together, these results suggest that incorporating spacers as part of Ab–NP conjugation enhances ligand conformational freedom and, by extension, promotes controlled orientation.

To help bolster stability under dynamic conditions while preserving the orientation associated with noncovalent biomolecular interactions, researchers have used “adapter” biomolecules such as biotin- or Fc-binding proteins (e.g., protein A or G), secondary Abs, oligonucleotides, or enzymes. These versatile adapter systems can rely on purely noncovalent interactions [15], although reactive chemistries may be used in conjunction to modify the NP surface, adapter, and/or Ab. Although such protocols are often expensive and require much optimization, they offer a straightforward approach to combining the advantages of different Ab–NP conjugation strategies [30]. Given the flexible nature of such proteins [85], it is possible that the same benefits provided by spacers translate to these adapters. Of particular interest is maintaining high bioactivity with densely packed, oriented Abs [86], which often requires a delicate balance between antigen binding capacity and selectivity [38], using adapter technologies [87,88]. That is, density and orientation may further represent a complicated tradeoff in preserving bioactivity [47,89], as confinement and the local environment directly influence Ab–receptor affinity [40]. Given their tunability and propensity for controlled surface attachment, adapters are uniquely suited to this challenge. Another possible avenue of exploration is covalent anchoring of Ab-compatible adapters to NP surfaces [86], although more research is needed to investigate whether strengthened adapter immobilization compromises biomolecular affinity. Such systems would combine the advantages of physisorption with the site specificity and environmental robustness of chemisorption. In all, adapter technologies that take advantage of biomolecular interactions, can be readily modified, and exhibit greater resistance to dynamic changes represent an intriguing area of study.

### Outlook for Ab–NP conjugation strategies

Given the superior potential of Ab–NPs over Abs alone (e.g., surface modifiability and drug loading) [37] or unconjugated NPs (e.g., targeting capability) [50], the connection between conjugate performance and Ab loading onto NP surfaces deserves greater attention. One innovative approach to improving existing conjugation strategies was demonstrated by microfluidics-assisted micromixing during chemisorption of anti-human epidermal growth factor receptor 2 (HER2) Abs onto PLGA/chitosan NPs. Escareño et al. [66] used a laminar flow regime to control Ab–NP interactions for optimization of coronal arrangement, as evaluated via NP size, zeta potential, and dispersity. Compared to classical bulk mixing, a microfluidics-based formulation enhanced drug release kinetics (with a faster release rate of doxorubicin) as well as uptake by and cytotoxicity in HER2^+^ breast cancer cells in vitro. The same conjugation chemistry, EDC/NHS coupling, was used in both cases, suggesting that mode of conjugation is not the only determinant of Ab-nanocarrier performance.

In addition to the mode of Ab conjugation, NP characteristics—including nanocarrier shape, size, and material—are important factors influencing Ab–NP performance. Barua et al. [90] compared polystyrene nano- and micro-spheres, -rods, and -disks to evaluate shape-based avidity of Ab-conjugated particles. Rod-like conjugates were found to have the greatest specific uptake and lowest nonspecific binding across multiple cell lines, likely due to higher surface area per unit volume that facilitated both better Ab loading and multivalent interactions with cells. Malaspina et al. [40] presented a theoretical argument for the advantage of smaller Ab–NPs: Smaller NPs have higher surface curvature, which strongly influences binding. Unlike larger particles that exhibit local planarity, NPs with smaller radii correspond to greater conformational entropy for conjugated Abs, allowing them to capture antigens more efficiently [40]. Related to nanocarrier shape and curvature is surface topology or texture, which helps regulate interfacial interactions (i.e., surface area and energy) with biological entities, including cells and proteins [91,92]. Modulation of surface topology is frequently accomplished via tuning PEG density [93–95], although PEG chain size and conformation can hinder ligand recognition and therefore target ability [94]. As surface topology influences protein physisorption [93,96], resulting changes in the protein binding affinity to NP surfaces determine conjugate pharmacokinetic performance in vivo [95]. Investigation into the role of nanoscale NP surface topology in Ab physisorption and, in particular, chemisorption processes could thus prove insightful.

It is also important to consider the material properties for specific applications, especially with respect to biocompatibility and biodegradability. Metallic NPs, for example, are quite small and tunable, but their neurotoxicity limits use of such materials in the CNS [97]. Fortunately, there are many materials that can be formulated into targeting nanocarriers, ranging from natural or synthetic polymers and liposomes to metal oxides or chalcogenides [56]. Organic–organic [70] and organic–inorganic [74,98] hybrid NPs for Ab conjugation represent an interesting avenue of exploration as well. The diversity of NP compositions complicates the relationship between NP properties and conjugation, as different materials require different surface immobilization approaches.

In an ideal system, immobilization of Abs onto NP surfaces would be highly site-specific, irreversible, oriented, and bioactive [38]. Hence, future investigations might benefit from focusing on reliable, facile control over Ab density and orientation. Density represents an interesting challenge, as oversaturation of NP surfaces with Abs can hinder cellular binding because of steric crowding [42]. Theoretical calculations indicate that increasing NP surface coverage of Abs improves antigen capture until these crowding effects greatly restrict ligand conformation, at which point the entropic penalty prevents additional antigen binding [40]. A key factor in the success of orientated attachment appears to be conjugation via the Fc region such that the antigen-binding sites protrude externally from the nanocarrier surface [30,36]. In this way, the Fc portion is less likely to interact with Fc receptors found in various cells throughout the body. Joshi et al. [99] achieved directional Ab immobilization to gold nanorods in this manner by oxidizing carbohydrate moieties in the Fc region of anti-epidermal growth factor receptor (EGFR) clone 225 or RG-16 Abs, chemically altering the gold surfaces via ligand exchange, and finally using a heterobifunctional cross-linker to conjugate the two. Similarly, Park et al. [49] employed deglycosylation at Asn^297^ in the heavy chains before introducing an azide moiety and reactive attachment to modified NP surfaces via strain-promoted alkyne–azide click chemistry. These methods demonstrate both the potential of site-specific, stable covalent conjugation of Abs in an oriented fashion and the need for adaptable, straightforward protocols. These studies of chemisorption-based conjugation via the Fc region reveal a need for further investigation, particularly regarding insights from subsequent evaluation in vivo.

One promising strategy for controlled ligand orientation is to use Ab fragments. Half Abs can be achieved using selective reduction of parent Abs via cleavage of disulfide bonds in the Fc region [68,72], thus generating reactively available sulfhydryl groups for subsequent covalent conjugation to maleimide-bearing NP surfaces. This method effectively halves the Ab size (and, by extension, the potential for undesirable interactions) while preserving site specificity and antigen-binding capability. Lourenço et al. [72] used thiol–maleimide click reactions to site-specifically conjugate half Ab fragments to polymer NPs in an oriented manner, preserving the antigen-binding site. Lee et al. [65] compared the performance of PLGA NPs conjugated to either full-length or F(ab′)_2_ fragments of CD8a Abs through EDC/NHS or maleimide–thiol chemistries, respectively. The latter had ~60% greater targeting efficiency at lower Ab or Ab fragment surface coverage (1 to 2 μg of Ab/mg of NP); similarly, for higher Ab content per particle (~5 to 8 μg/mg), Ab fragments outperformed full-length Abs by ~20% to 30% [65]. They attributed this phenomenon to the random versus controlled orientations that characterized immobilization of the full-length Abs or Ab fragments, respectively. Although Ab fragments may have lower stability, possibly due to loss of bioactivity upon reduction and packing-based steric hindrance, they provide a reasonably facile route to oriented immobilization via site-specific chemisorption [100].

Most of the prior research discussed in this section involved in vitro work, using cultured cells, which is typically performed in a noncompetitive environment with well-controlled variables and uniform accessibility to cellular targets. Conversely, receptor–ligand interactions in vivo face heterogeneous concentrations, competition from local interactions, and ever-changing binding conditions [26]. Use of inert polymer coatings can help shield targeting NPs from undesired interactions and aggregation [50], extending circulation time without substantially reducing adsorption [101]. However, these modifications can require multistep chemistries [74] and induce protein denaturation or orientational changes that decrease immunoactivity [101]. Such remedies are system-specific, making them difficult to adapt across materials, conjugation protocols, and environmental conditions. Although in vitro data provide important complementary information [51], complicating and unpredictable factors in vivo often translate to discrepancies in performance [102].

Many of the prior investigations into Ab–NP conjugates focuses on biosensing applications [39,45,47,68,69,103], but the tunability and targeting ability of such systems suggest a potential in therapeutics. Future investigations could thus explore the influence of conjugation strategy (e.g., optimization for detection as opposed to treatment) to compare ideal Ab–NP qualities for sensing versus for therapy. For nanomedicine applications, Ab−NPs should maintain bioactivity, exhibit stealth behavior, and achieve prolonged contact with targeted cellular populations whilst avoiding off-target binding in vivo [26]. Therefore, it would be useful to devote more effort to investigating Ab–NPs in a dynamic, nonequilibrium environment. Such research could more explicitly elucidate the relationships between Ab−NP conjugation and performance in vivo to reveal the most important design elements (e.g., interaction strength or ligand density) in conjugate preparation.

## Ab–NP Conjugates for Targeting Disease in the CNS

Ab−NP conjugates can target biomarkers that are overexpressed in diseased cells but are expressed at low levels by healthy cells [35]. Many therapeutic efforts with Ab–NPs focus on cancer and on improving survival in vivo, by targeting EGFR [104], CD44 [105], mucin 1 C terminus [106], HER2 [107] (which are overexpressed in a variety of cancers such as breast and lung cancers), and death receptor 5 [108] (which are overexpressed in pancreatic cancer). While numerous therapeutic Ab–NP formulations demonstrate potential in vitro, they often face considerable challenges in vivo, due to the complex and dynamic nature of physiological systems [37,109]. Several of these obstacles are now well described: clearance by the reticuloendothelial system, difficulty in entering dense tumor tissues, and high interstitial pressure in tumors, which expels therapeutic agents [110]. Targeting cellular populations within the CNS further presents a unique barrier, as Ab–NPs must overcome the BBB before interacting with target cells. Understanding of the factors that govern conjugate performance in vivo—namely, cellular interactions with NPs and the effect of Ab–NP conjugation—is crucial to the success of these therapeutic tools. Recent efforts for systematic evaluation [35,111] provide new insights into Ab–NP combinations that might be most successful in animal or clinical studies. However, more work is still needed to identify the most influential design considerations for Ab–NP conjugate performance in vivo.

### Lessons from systemically delivered Ab–NP conjugates

Developing Ab–NP systems requires consideration of myriad design aspects and their influences on conjugate performance. A recent meta-analysis suggests that incorporation of Abs on NP surfaces improves targeted delivery of the injected dose, when compared to bare or unconjugated NPs [35]. NP size and composition appear to modulate this effect: The smaller the size of the Ab–NPs, the better the tumor uptake. On the other hand, pharmacokinetics and biodistribution of Ab–NPs generally appear similar to those associated with bare NPs. This finding aligns with another recent study demonstrating that the nature of core NP material exerts more influence over NP–cell interactions for uptake than surface chemistry, which more subtly influenced these interactions [111]. However, not all Ab–NPs follow these trends. A closer examination of individual studies suggests a more nuanced relationship between Ab–NP design factors—including ligand density, affinity, and valency—and conjugate targeting ability, particularly for systemic delivery. While most of the following studies involve targeting of extracranial tumors, evaluating these design components may reveal key elements needed for CNS targeting by Ab–NP systems.

Ab density plays an important role in improving conjugate targeting; modulation of Ab surface packing can result in pharmacokinetic penalties that cannot be fully characterized with in vitro studies. Colombo et al. [102] found that conjugating a single trastuzumab Ab to each 5-nm spherical gold NPs led to more efficient active targeting in vivo than conjugating two Abs. This finding contradicted the trend in vitro, where an increased number of conjugated Abs increased uptake in cell lines. The authors concluded that the moderate improvement in targeting with two Abs per NP was negligible compared to the increased reticuloendothelial system uptake in vivo that resulted from the corresponding increase in NP size (2.4× for one Ab/NP, 4× for two Abs/NP) [102]. Even for NP systems that maintain comparable sizes, increasing ligand attachments on NP surfaces can impair biodistribution and pharmacokinetics, as nonspecific protein absorption for clearance and unspecific uptake also increase [110]. While an optimal Ab density for improved uptake was observed in vitro, Reuter et al. [16] found that all Ab-conjugated hydrogel NPs, regardless of Ab density, had increased clearance and shorter half-lives than PEGylated controls in vivo. With minimal shift in size from Ab attachment (i.e., no more than a 20-nm shift in size for 80-nm × 320-nm rod-like NPs and no appreciable shift for 55-nm × 60-nm spherical NPs), the contribution of “passive” targeting was ultimately more substantial than “active” targeting in this situation.

Maintaining NP size may help realize the “active” targeting qualities of Abs in vivo. With no notable shifts in NP size and biodistribution observed, Rodallec et al. [112] evaluated Ab density via trastuzumab conjugated on drug-loaded immunoliposomes—with 330, 480, or 690 Ab molecules per 140-nm liposome—and found that in vitro the moderately dense immunoliposomes (480 Abs per liposome) led to the greatest specific uptake. The moderately dense immunoliposomes also proved efficacious in vivo [113]. Similarly, Jain et al. [114] maintained size similarity while incorporating anti-mesothelin Abs into drug-loaded 4.5G poly(propylene imine) dendrimers, with average diameters of <100 nm, which slightly improved the pharmacokinetics and demonstrated therapeutic efficacy.

Increasing Ab–NP conjugate size does not always yield poor pharmacokinetics. Using Si–Gd NPs of similar sizes (4.4 nm [115] and 6.3 nm [106]), Detappe et al. found that Ab–NP complexes (~2.3 to 2.9× size increase per NP) had a greater circulatory half-life than unconjugated NPs, as well as prolonged contact with tumors. However, these nanocarriers were found to consist of four NPs on average bound to a single Ab [106]. Considered together, these findings suggest that Ab surface density and NP size play important roles in “active targeting”, although further work is needed to clarify this balance for both nanocarriers averaging less than one Ab each and, more commonly, Ab–decorated NPs on which this review focuses.

Additional evidence suggests that active targeting with Abs can also be optimized by adjusting affinity and valency, rather than density. In one example that targeted the CNS, Johnsen et al. [116] labeled 50-nm gold NPs thoroughly with an anti-transferrin (TfR) Ab and compared different affinities and valency. Abs exhibiting very low affinity moderately outperformed those with higher affinity. Modulating valency, however, generated markedly different results: A bispecific/monovalent Ab with higher affinity performed much better than the bivalent Ab with reduced affinity. These findings suggest that exploiting specific Ab–target interactions, whether through Ab monovalency or by reduced affinity, can lead to more effective active targeting. Johnsen et al. [116] discussed previous in vitro work from Gregori et al. [117] that evaluated Ab affinities, which found that higher-affinity anti- TfR Abs (*K*_D_ = 14.5 nM) performed better than those with lower affinity (*K*_D_ = 64.3 nM) in immunoliposomes. However, this prior work used Abs with dissociation constants that are much smaller and, therefore, have much higher affinity than that used by Johnsen et al., who measured the *K*_D_ of their own low affinity as 149 nM. Also intriguing is that 40 to 45 Abs were calculated to be on ~100-nm immunoliposome of Gregori et al., while Johnsen et al. calculated 7 to 9 Abs per ~80-nm Au NP; consideration of ligand density may also be needed to shed further light on how affinity and valency can best realize the targeting efficacy of Abs in the CNS.

Two alternative strategies for developing effective Ab–NP merit mention using smaller Ab fragments for targeting and harnessing the targeting capacity of the immune system. First, the use of Ab fragments may lead to preservation of favorable pharmacokinetics while endowing NPs with improved targeting ability, as described above [65]. Cheng and Allen [118] performed a thorough pharmacokinetic evaluation with liposomes, which demonstrated the longer circulation half-life (18.1 h versus 5.6 h), improved targeting capability, and better survival outcomes of Fab′-conjugated immunoliposomes compared to those with full-length Ab. More recently, Duan et al. [119] showed that Fab′ fragments of trastuzumab to PEG–PLGA NPs led to superior circulation half-lives (3.0× longer than with full-length Ab–NPs and 5.3× longer than with bare NPs) and tumor targeting (~16% more than with full-length Ab–NPs). The advantages of Ab fragments over full-length Abs may depend on material; Mittelheisser et al. [35] suggested through their meta-analysis that polymeric, inorganic, and organic NPs (the latter two placed into the same category for analysis) benefit more from full-length Abs, while Ab fragments are more advantageous in liposomes. However, these trends might not be generalizable because of the relative lack of available data used in the meta-analysis: 161 full Ab–NP data points versus 18 Ab fragment–NP data points.

Second, instead of targeting a disease directly, Ab–NPs that target relevant immune cells may offer greater specificity and therapeutic effect. A main challenge in immunotherapies is addressing the immunosuppression of the tumor microenvironment. To combat this, Zheng et al. [120] demonstrated tumor regression in mouse models for adoptive cell therapy using Ab–NPs that deliver specifically to donor T cells in vivo a payload of free transforming growth factor-β inhibitors. By targeting a specific isoform of a receptor in donor T cells with conjugated Abs, Ab–NPs indirectly localized to the tumor, delivered the transforming growth factor-β inhibitors within, and reverted the immunosuppressive effect on the T cells. Targeting immunosuppressive cell populations can also be a viable strategy. In a novel two-step approach, Lee et al. [121] first administered an Ab for CD11b, modified with a *trans*-cyclooctene, which targets immature myeloid cells that infiltrate deep into tumors and develop into tumor-associated macrophages. Second, the authors administered doxorubicin-loaded mesoporous silica NPs bearing 1,2,4,5-tetrazine, which specifically binds to the modified CD11b Ab. This strategy exploited tumor-recruited immune cells to deliver a depot of drugs deep into the tumor mass and enhance therapeutic efficacy in vivo. A burgeoning field, the synergy between Ab–NPs and cells of the immune system, holds great therapeutic promise, particularly since it builds on prior knowledge of antigen targets that characterize specific populations of immune cells.

### Crossing the BBB

**Fig. 5.**
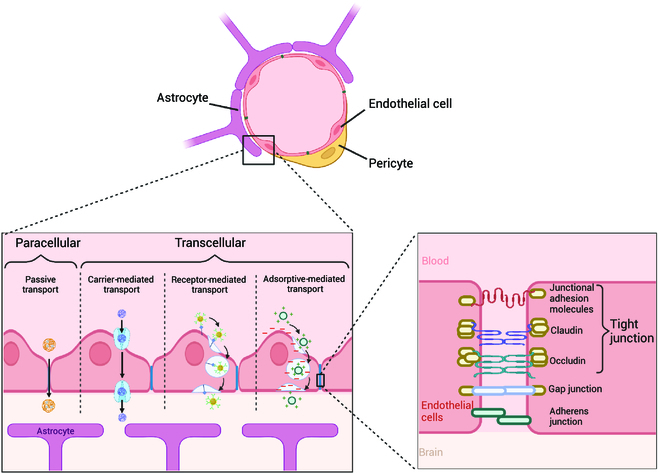
Transport mechanisms of NPs across the BBB. There are three types of junctional complexes in the BBB: tight junctions, adherens junctions, and gap junctions [185,186]. Tight junctions prevent most molecules—and NPs—from easily crossing the barrier. Created with BioRender.com.

The BBB is a selectively permeable microvasculature structure that protects the CNS and helps maintain the delicate environment in which the CNS functions. Tight junctions between endothelial cells along the BBB facilitate highly regulated transport of molecules into the brain [10,25,122,123]. Molecules and other vehicles that do pass through the BBB via these junctions use specific pathways for paracellular transport or transcellular transport. Transcellular pathways are of particular interest for NPs, as their small size can allow them to exploit carrier-mediated transport, receptor-mediated transcytosis, and adsorptive-mediated transcytosis; however, in some cases, NPs are also able to passively diffuse across paracellular pathways (Fig. 5) [10]. Alternatively, NPs can be paired with physical approaches that overcome the BBB, either by creating local regions of transiently higher BBB permeability with FUS or by direct infusion of NPs into the brain parenchyma by CED. Because of their targeting properties, it has been speculated that Ab–NPs can capitalize on receptor-mediated transcytosis, which requires a cellular interaction with a receptor on the endothelium (Fig. 5).

The most commonly exploited target for Abs to enter the CNS is TfR, which carries iron across the BBB. Anti-TfR-conjugated poly(lactic acid) NPs [124,125], PEG–poly(lactic acid) NPs [126], dendrimers [127], gold NPs [116], mesoporous silica NPs [128], liposomes [129], human serum albumin NPs [130], and QDs [131,132] have been reported to cross the BBB. A variety of methods have been used to confirm successful crossing, such as in vivo imaging (e.g., single-photon emission computed tomography [124]), analysis of isolated cell populations in the brain (e.g., counting of NPs after harvesting and dissociating brain tissue [128,129,131]), in vitro BBB models [126,127], or evaluation of a unique function of the delivered payload effect in vivo (e.g., reduced nociception through opioid agonist delivery [130]). However, targeting TfR has limitations; TfR expression decreases after administration of bivalent anti-TfR Ab constructs [133–135], while monovalent constructs maintain the same level of expression. Thus, for therapies that require repeat dosing, a reduced TfR target binding may be crucial.

Insulin receptors (InR) have also been used as targets for Ab–NP transport across the BBB. The InR Ab 29B4 was covalently conjugated to human serum albumin NPs; the resulting conjugates were able to pass through the BBB and elicit therapeutic behavior [130]. In another example, intravenous administration of liposomes with covalently coupled InR Ab 83-14 was shown to deliver an encapsulated plasmid, which was transiently expressed in cells in the brain of nonhuman primates [136]. These studies build upon ADC strategies, which have led to in vivo brain imaging [137] and therapy for cognitive stabilization [138], using a human InR monoclonal Ab that crosses the BBB via receptor-mediated transcytosis.

In addition to TfR and InRs, other targets for receptor-mediated transcytosis in the BBB are already well established, such as low-density lipoprotein receptor-related protein 1 (LRP1) in the low-density lipoprotein receptor (LDLR) family [10,139] and choline receptors [140]. However, most are being pursued primarily with peptides or substrate molecules, which are smaller than full-length Abs and demonstrate desirable qualities such as nonimmunogenicity and improved binding with receptors [141]. Ab fragments provide similar advantages, while maintaining the active targeting aspects of full-length Abs [32]. Consequently, the field may benefit from a focus on Ab fragments, or other antigen-binding proteins, rather than full-length Abs, for overcoming the BBB. Other strategies for improved NP penetration of the BBB—which do not involve Ab or Ab fragments—have also been reported, such as stimulus response [142,143] or incorporation of cell-penetrating peptides [144–147] or surfactants [148–150] on the NP surface.

Abs can also facilitate NP penetration of the BBB by first specifically labeling the BBB surface before undergoing conjugation to NP surfaces in vivo. Gonzalez-Carter et al. [151] used biotinylated anti-platelet endothelial cell adhesion molecule (PECAM) Abs to label the BBB before administering avidin-conjugated nanomicelles. Biomolecular interactions between the biotinylated Abs and avidin-coated nanomicelles then facilitated Ab–NP conjugation and subsequent crossing of the BBB. This strategy reduced off-target uptake—which, in this case, would occur in other PECAM-expressing tissues such as heart, lung, and pancreas—by leveraging differential endocytic rates between organs; as the endocytic turnover of PECAM is lower at the BBB compared to the other organs, the time interval between primary administration of the Ab and secondary administration of the nanomicelles could be optimized such that the biotin targets only remained on the BBB. By evaluating differential endocytic rates between “target” and “off-target” organs, especially since common targets for the brain such as TfR1 and LRP1 are highly expressed in other organs, specific uptake through the BBB can be improved.

Measurement of Ab–NP transport across the BBB is not straightforward; identifying the individual impact of NP distribution (i.e., BBB transport) and drug distribution in the brain is crucial [152]. While perhaps the most persuasive measure of the penetration of the BBB is the observation of therapeutic effects in animal models of CNS disease, effectiveness does not necessarily indicate successful BBB penetration; for example, effectiveness might be due to Ab-mediated retention on the luminal surface of the BBB and subsequent release and transport of an encapsulated drug. Incorporating other analytical methods, most convincingly imaging, is important to link effectiveness of the Ab–NP with BBB penetration. The use of complementary imaging methods in vivo is especially compelling, although this often requires the incorporation of other elements that allow for imaging (e.g., fluorophores) into the NP design [124,125,129]. These added elements also create opportunities for artifacts (such as through imaging probe detachment) that may make experimental results difficult to interpret [153]. Alternative quantitative approaches to evaluating Ab–NP transport across the BBB include pharmacokinetic measurements and biodistribution studies, in which radioisotopes are often incorporated [154]. Still, the lack of conclusive methods for characterizing transport of unmodified Ab–NPs across the BBB remains a challenge for the field.

### Targeting cellular populations in the CNS

Abs have long been explored as therapeutics for cancer because of their ability to bind to specific disease targets [155]. For example, trastuzumab has been approved for clinical use since 1998, where it is used to treat breast cancer by targeting cells that express the HER2 protein. Abs can also help NPs to target cancerous cellular populations in the CNS. In this setting, trastuzumab has been extensively incorporated as an active targeting ligand on NPs, particularly cancer cells that have metastasized into the CNS from a peripheral HER2^+^ cancer [156]. For example, He et al. [157] explored a two-step targeting nanoplatform, where a self-assembled nanoconstruct of polysorbate 80-conjugated polymer, lipids, and polymer-conjugated trastuzumab was administered systemically. In this case, polysorbate 80, a small molecule, facilitated the BBB crossing through LDLR-mediated transcytosis. The NP complex was to then accumulate into the tumor through the EPR effect—although it has not been shown to be relevant in brain tumors [17–20]—and subsequently dissociate over time. This approach allowed the released trastuzumab-polymer component to target the HER2^+^ cancer cells in the brain, resulting in enhanced therapeutic effects compared to free drug. Meanwhile Ngamcherdtrakul et al. [158] delivered PEGylated mesoporous silica NPs conjugated with trastuzumab, which released electrostatically bound small interfering RNA and encapsulated docetaxel to the tumors. In this case, the NP was delivered in the setting of FUS opening of the BBB, where it appeared to be safe and effective in mice with breast cancer metastasis to the brain.

Additional brain tumor targets include EGFR, CD133, and tumor-specific nucleosomes. CED of cetuximab-conjugated iron oxide NPs demonstrated therapeutic effects in vivo for both EGFR-expressing glioblastoma (GBM) xenografts in mice [159] and spontaneous intracranial gliomas in dogs [160]. Temozolomide, an oral chemotherapy drug that is frequently used to treat GBM, was delivered for therapeutic effects to GBM stem cells through a liposome conjugated to both angiopep-2, a peptide that transcytoses the BBB through LRP1 binding, and CD133, a biomarker for glioma stem cells (a subset of tumor cells that display high resistance to chemotherapy and radiation therapy) [161]. The anti-nucleosome Ab 2C5 has also been used with a TfR Ab on a poly(β-l-malic-acid) nanoplatform to target GBM [162]. In these examples, there are two strategies at work simultaneously: penetrating the BBB and targeting of cancer biomarkers to enhance the therapeutic effects.

Immune cells have also served as targets in the CNS. Dendrimers conjugated to cell-penetrating peptide tLyp-1 and anti-natural killer group 2a (NKG2a) Abs have shown efficacy in vivo, the former promoting receptor-mediated transport across the BBB and the latter facilitating T and natural killer cell activation against tumors [163]. Other conjugates used to target immune cells incorporate immune checkpoint inhibitors—Abs against cytotoxic T-lymphocyte-associated protein 4 and programmed cell death protein 1—which increase T cell, natural killer cell, and macrophage activity in the vicinity of the brain tumor. In one example, Galstyan et al. [164] used a poly(β-l-malic-acid) NP, simultaneously attached to TfR Abs for BBB crossing and Abs targeting immune cells local to the tumor.

Native CNS cellular populations have also been targeted using Ab–NPs, as well as NP-only platforms that could be developed into Ab–NP systems. Neurons have been targeted by incorporating Abs against highly expressed ion channels: Upon direct injection into the brain, photoacoustic NPs bearing Abs against the ion channel transient receptor potential cation channel subfamily V member 4 (TRPV4) attached the to neurons and facilitated neuronal stimulation in vivo [165], as did PEGylated polydopamine-coated gold NPs with Abs against ion channel TRPV1 [166]. Other platforms, such as squalenoyl adenosine NPs [167], lipid NPs [168], and iron oxide NPs conjugated to peptide cargo [169], have demonstrated successful uptake into neurons in vivo and could incorporate Abs for greater specificity. Neuroblasts have also been targeted to support their potential differentiation and maturation to neurons: intracranial administration of porous silicon NPs conjugated to anti-polysialylated neural cell adhesion molecule Abs were able to reach neuroblasts and promote proliferation signaling [170].

As discussed earlier, Ab–NPs can target the endothelial cells that comprise the BBB, either to promote localization for selective release [151] or to facilitate receptor-mediated transcytosis. Oligodendrocyte precursors, which express neuron-glial antigen 2 (NG-2), have been targeted by PLGA NPs with conjugated anti-NG-2 Abs. These Ab–NPs delivered leukemia inhibitory factor, to encourage remyelination within the context of multiple sclerosis [171]. Microglial cells and astrocytes have also been successfully targeted by both NPs and Ab–NPs. These cellular populations are biological targets of particular interest due to their role in inflammation and neurodegeneration. Modulating their activity can generate therapeutic effects, depending on the disease. Silver NPs delivered intranasally, for instance, have been found to increase microglial activation in rats [172], while human mesenchymal-stem-cell-derived exosomes reduced microglial activation in rats with perinatal brain injury [173]. When systemically delivered, certain dendrimers have been observed to cross the BBB, accumulate in the microglia and astrocytes of rabbits with cerebral palsy, but not in healthy animals [174], and to mediate delivery of drugs [175]. Although few Abs have been used yet in combination with NPs to target these cellular populations, Ab–NPs have shown promise; for example, anti-CX3CR1 and anti-CD11b are two microglia-targeting Abs incorporated into NPs (PLGA [176] or ceria-zirconia [177], respectively) that demonstrated success in addressing neuropathic pain. Furthermore, previous work has shown that surface incorporation of non-Ab ligands, either to enhance BBB crossing or to target specific tissues, can lead to successful NP uptake by microglia and neurons [142,143,148,178]. Hence, future studies may build upon the ability of Ab–NPs to target these cells and therefore reduce neurotoxicity or off-target effects.

It is also worthwhile to note that, often, Ab–NPs targeting specific neuronal populations are combined with local administration methods, such as intrathecal [176,177], intranasal [172], and intracranial [171] delivery. Although some comprehensive efforts have been made [179], additional investigations may reveal how route of administration influences the targeting capacity of Ab–NPs.

### Outlook

Balancing ligand density, affinity, and/or valency to improve active targeting is not intuitive, especially given the sometimes conflicting in vitro and in vivo observed to date. For example, the “less is more” approach of enhancing specificity by reducing some aspect of Ab is also reflected in dosage of Ab−NPs: Singh et al. [180] found that active targeting is fully realized with lower Ab−NP amounts in vivo. At high doses (12 mg/kg), Ab−NPs and control NPs demonstrated similar therapeutic efficacies; lower doses of Ab−NPs (2 mg/kg), on the other hand, revealed improvement from the Ab–NPs over the controls. Balancing effective dosing with improved targeting requires thoughtful and careful design, just as Ab−NP conjugate design must balance attributes of the NP and the Ab (or Ab fragment) [116].

For the success of future Ab−NPs that target the CNS, their design must consider and implement understanding of how targeting mechanisms operate in vivo. Recent work has explored interactions between the host immune system and NPs. Using an in vivo breast cancer model, Korangath et al. [181] recently demonstrated that conjugation of hydroxyethyl-starch-covered iron oxide NPs to trastuzumab increases accumulation in the tumor regardless of HER2 expression—in contrast to their in vitro effectiveness, which required HER2 expression. In fact, the systematically administered NPs inhibited tumor growth regardless of Ab conjugation [181]. It appears that NPs localized to tumor-associated immune cells more in mice with intact immune cells than compromised, which indicates a more complex role of the immune system in NP biodistribution and effectiveness. This lack of an ability to predict NP effectiveness in vivo based on in vitro measurements is a major impediment to progress in the field.

A greater understanding of how nanomaterial surfaces—as well as any associated protein corona—interact with cells in their natural setting in intact tissues is crucial for progress. A recent large screening of 35 NP types across 488 cancer cell lines revealed biomarkers and specific cellular interactions that were important in determining NP uptake [111]. Material and surface coating affected the number and identity of biomarkers, and incorporation of these considerations in rational NP design successfully predicted their in vitro and in vivo behavior. As the field continues to clarify how NPs are effectively taken up by cells, rational design of CNS-targeting Ab-NPs will require further studies of this type, providing insight into factors that will allow prediction of in vivo performance from in vitro (or even in silico) experiments.

Last, crossing the BBB and targeting cellular populations in the CNS present at least two different obstacles for Ab–NPs, which suggests a need for multiple functions in one platform. While some studies have coupled physical approaches to crossing the BBB with cellular-targeting Ab–NPs [158,160,182], other efforts have focused on conjugating multitargeting Abs onto one nanocarrier. For example, Fujita et al. [162] found that such a platform yielded better outcomes with two different Abs in tandem—anti-TfR and anti-nucleosome 2C5—than single Abs alone. Similarly, Johnsen et al. [116] found that a bispecific Ab, anti-TfR/ beta-site amyloid precursor protein cleaving enzyme 1 (BACE1), outperformed monovalent Abs in crossing the BBB, although a therapeutic effect of BACE1 was not observed. At the same time, the use of smaller Ab fragments, or other antigen binding proteins [183], may have several advantages: preventing the unfavorable shift in pharmacokinetics observed with NPs conjugated to full-length Abs, promoting controlled orientation, and maintaining targeted binding.

## Conclusion

A major factor in the translation of Ab–NPs to therapeutic applications is controlled immobilization of targeting Abs on tunable NP surfaces. Two primary modes of conjugation, physisorption and chemisorption, have each been explored at length for such attachment [15,30,38]. Conjugation strategies can directly influence Ab–NP behavior [40,43,72]; generating effective therapies based on Ab–NPs will depend on developing appropriate attachment techniques. In the CNS, biocompatibility and enhanced contact of therapeutic nanocarriers with targeted cellular populations are especially important considerations [184]. In vivo studies comparing different immobilization methods—using the same NP and same Ab, but a different conjugation method—would be extremely informative. However, few investigations have sought to compare Ab–NP conjugation method and performance in the CNS. Ideal conjugation protocols would combine the attributes of physisorption (controlled orientation, preservation of bioactivity, and simplicity) with those of chemisorption (site specificity, insensitivity to dynamic changes, and bond strength).

Oriented, robust immobilization of Abs onto NP surfaces is a key metric of successful conjugation [36]. There are fortunately several promising avenues to pursue in defining the best methods for conjugation, but more in vivo work is needed to determine the viability of such conjugates for clinical translation. Much of the existing research into conjugation strategies focuses on detection over therapeutic treatment [39,68,103] and relies primarily on in vitro work [49,72,99]. Additional in vivo studies might more explicitly define the relationship between conjugation and Ab-NP conjugate behavior, particularly as there appear to be inconsistencies between in vitro and in vivo performance [37]. Given the vast parameter space as yet unexplored in Ab–NP conjugation, many opportunities exist to expand the current repertoire and, by extension, to inform the design of effective Ab–NP therapies.

Although a recent meta-analysis of Ab-NPs concluded that pharmacokinetics are primarily determined by NP properties, rather than Ab moieties, conjugation method is relatively unexplored in vivo [35]. Furthermore, the effects of Ab density, affinity, and valency on pharmacokinetics and targeting suggest that more systemic investigations are required. Clarifying how these underexplored aspects, in addition to the well-considered parameters of NP size and material, influence in vivo performance will be especially useful in future conjugate design. Moving forward, such improved understanding may help elucidate the advantages of Ab fragments—which offer smaller sizes, less immunogenicity, more precise and oriented attachments, and enhanced antigen-binding capability—over full-length Abs.

Developing effective Ab–NPs for treating CNS disorders requires addressing many challenges simultaneously. Emerging strategies appear to capitalize on several different interactions simultaneously: Just as multistep chemistries have been shown to endow site-specific stable, oriented, covalent conjugation of Abs to NPs [49,99], multitargeting Ab–NPs [162] may generate nanoplatforms that overcome the BBB and target a specific cellular population in the CNS. Currently, multistep ligand–NP mechanisms for therapeutic effects have been explored for small peptides [140] and have been preliminarily evaluated for Abs [151]. At an even larger scale, combining multiple targeting systems, such as Ab–NPs that target immune cell, which hone to the disease, may hold even greater potential [181].

Ab–NPs represent a versatile platform with great promise for clinical translation. Rational design of Ab–NPs will be crucial to maximizing their potential impact in the CNS.
